# Kinetic modelling of quantitative proteome data predicts metabolic reprogramming of liver cancer

**DOI:** 10.1038/s41416-019-0659-3

**Published:** 2019-12-10

**Authors:** Nikolaus Berndt, Antje Egners, Guido Mastrobuoni, Olga Vvedenskaya, Athanassios Fragoulis, Aurélien Dugourd, Sascha Bulik, Matthias Pietzke, Chris Bielow, Rob van Gassel, Steven W. Olde Damink, Merve Erdem, Julio Saez-Rodriguez, Hermann-Georg Holzhütter, Stefan Kempa, Thorsten Cramer

**Affiliations:** 1 Charité – Universitätsmedizin Berlin, corporate member of Freie Universität Berlin, Humboldt-Universität zu Berlin, and Berlin Institute of Health, Institute of Biochemistry, 10117 Berlin, Germany; 2 Charité – Universitätsmedizin Berlin, corporate member of Freie Universität Berlin, Humboldt-Universität zu Berlin, and Berlin Institute of Health, Institute for Computational and Imaging Science in Cardiovascular Medicine, 13353 Berlin, Germany; 3 Molecular Tumor Biology, Department of General, Visceral and Transplantation Surgery, RWTH University Hospital, 52074 Aachen, Germany; 4 Berlin Institute for Medical Systems Biology, Max-Delbrück-Center for Molecular Medicine, 10115 Berlin, Germany; 5 Joint Research Centre for Computational Biomedicine, Faculty of Medicine, RWTH Aachen University, 52074 Aachen, Germany; 6 Bioinformatics Solution Center, Freie Universität Berlin, 14195 Berlin, Germany; 7 Department of Surgery, Maastricht University Medical Centre, 6229 ER Maastricht, The Netherlands; 8 NUTRIM School of Nutrition and Translational Research in Metabolism, Maastricht University, 6229 ER Maastricht, The Netherlands; 9 Institute for Liver and Digestive Health, University College London, London, WC1 6HX UK; 10 ESCAM – European Surgery Center, Aachen, Germany; 11 ESCAM – European Surgery Center, Maastricht, The Netherlands

**Keywords:** Cancer metabolism, Hepatocellular carcinoma, Computer modelling

## Abstract

**Background:**

Metabolic alterations can serve as targets for diagnosis and cancer therapy. Due to the highly complex regulation of cellular metabolism, definite identification of metabolic pathway alterations remains challenging and requires sophisticated experimentation.

**Methods:**

We applied a comprehensive kinetic model of the central carbon metabolism (CCM) to characterise metabolic reprogramming in murine liver cancer.

**Results:**

We show that relative differences of protein abundances of metabolic enzymes obtained by mass spectrometry can be used to assess their maximal velocity values. Model simulations predicted tumour-specific alterations of various components of the CCM, a selected number of which were subsequently verified by in vitro and in vivo experiments. Furthermore, we demonstrate the ability of the kinetic model to identify metabolic pathways whose inhibition results in selective tumour cell killing.

**Conclusions:**

Our systems biology approach establishes that combining cellular experimentation with computer simulations of physiology-based metabolic models enables a comprehensive understanding of deregulated energetics in cancer. We propose that modelling proteomics data from human HCC with our approach will enable an individualised metabolic profiling of tumours and predictions of the efficacy of drug therapies targeting specific metabolic pathways.

## Background

Numerous initiatives around the world in conjunction with the unprecedented development of high-throughput analytical methodology have spiralled up the molecular knowledge about cancer in dizzying heights.^1^ Clinical translation of the newly gained information has resulted in the approval of a plethora of molecular-targeted drugs with anti-proliferative activity. However, despite all these advances, the overall death rate for cancer has declined at a much slower pace in the past 40 years compared to other major causes of mortality such as cardiovascular and infectious diseases.^2^ This is, to a large extent, explained by suboptimal long-term anti-proliferative efficacy of newly developed molecular-targeted drugs.^3^ Cancer cells display a marked capability to compensate for the inactivation of signalling pathways—and other growth-promoting mechanisms—that are considered essential for neoplastic progression.^4,5^ This translates into the emergence of therapy resistance, a major obstacle of clinical oncology.^6^ To achieve effective and long-lasting therapy responses it is therefore of pivotal importance to identify processes that are at the same time essential and unique, thereby avoiding resistance via usage of alternative pathways. Metabolism represents such a process as it is essential for cellular survival and growth, e.g. by providing energy, reductive power and building blocks for anabolic reactions. In light of the uniqueness of metabolic pathways, their functional inactivation can usually not be compensated, resulting in robust cellular damage and/or death.^7^

The notion that tumours display specific metabolic alterations that can be exploited for diagnosis and therapy of cancer has received widespread attention in recent years.^8^ However, it also became evident that a reliable analysis of metabolism, especially under in vivo situations, is challenging to perform.^9^ This is due to various factors, such as the rapid turnover of substrates and products, the intricate complexity of the metabolic network, the central importance of external stimuli such as hormones, growth factors and the cellular microenvironment.^10^ One main reason for the insufficient understanding of metabolic changes in tumours is the strong focus on changes in the expression level of metabolic enzymes and transporters. The importance of downstream kinetic regulation, for instance by allosteric effects or reversible phosphorylation, has been underestimated or completely disregarded in the last two decades.^11^ Choosing glucose metabolism of the liver as an example, we have recently demonstrated the necessity to combine existing knowledge on gene expression changes with the complex kinetic regulation of enzymes in order to understand the metabolic response of the liver to varying external challenges.^12^ Here, we present an innovative concerted approach to study cancer metabolism by combining a novel physiology-based kinetic model of the central metabolism,^13^ with high-quality quantitative proteomics data and molecular biological experimentation to elucidate metabolic differences between liver cancer (hepatocellular carcinoma, HCC) and the normal liver in a murine model. Using relative changes in the expression level of metabolic enzymes in HCC and normal liver cells to scale maximal enzyme activities, we simulate the metabolic response of HCC and the normal liver to variations in the metabolite and hormone profile of the blood plasma. This enables the definition of conditions at which the metabolism of the tumour becomes severely impaired while the metabolism of normal liver cells remains largely unaffected. We strongly believe that the herewith presented approach bears translational potential and will outline a basic roadmap to achieve this.

## Methods

### Transgenic HCC Model and tissue preparation

The murine HCC model (termed ASV-B; C57Bl/6 J background) was established and initially characterised by Dubois and co-workers.^14^ Briefly, male ASV-B mice express the early region of the SV40 large T (SV40lT) oncogene under control of the mouse antithrombin III promoter. ASV-B mice show time-dependent liver tumour development with first evidence of dysplasia at 8 weeks, adenomas at 12 weeks and hepatocellular carcinoma (HCC) at 16 weeks of age. All mice were maintained under specific pathogen-free conditions under a 12 h light/dark cycle with free access to food and drinking water and cage enrichment at the animal facilities in Berlin (Charité) and Aachen (Institut für Versuchstierkunde, University Hospital Aachen). Animal procedures were performed in accordance to approved protocols (Landesamt für Gesundheit und Soziales Berlin and Landesamt für Natur, Umwelt und Verbraucherschutz Recklinghausen) and followed recommendations for proper care and use of laboratory animals. For tissue preparation, 16-weeks-old ASV-B or tumour-free male control mice (C57Bl/6 J, Harlan Laboratories) were sacrificed by cervical dislocation and liver tissue samples were snap-frozen in liquid nitrogen. Further analyses of tissue samples were performed within 2–4 weeks.

### Metabolic model

We have recently developed a comprehensive kinetic model of the central metabolism of hepatocytes.^13^ The model was used to simulate the impact of nutrient supply (including oxygen), hormonal stimuli and protein abundance of metabolic enzymes on the functional output of the liver. The model comprises the central hepatic metabolic pathways of: glycolysis, gluconeogenesis, glycogen synthesis, glycogenolysis, fructose metabolism, galactose metabolism, the creatine-phosphate/ATP shuttle system, the pentose phosphate cycle composed of the oxidative and non-oxidative branch, the citric acid cycle, the malate aspartate redox shuttle, the glycerol-3-phosphate redox shuttle, the mitochondrial respiratory chain, the beta-oxidation of fatty acids, fatty acid synthesis, ketone body synthesis, cholesterol synthesis, triglyceride synthesis and degradation, the synthesis and hydrolysis of triglycerides, the synthesis and export of the very-low density lipoprotein (Vldl), the urea cycle, the metabolism of the amino acids serine, alanine, glutamate, glutamine, aspartate and ethanol metabolism. Furthermore, the model contains the key electrophysiological processes of the inner mitochondrial membrane including the mitochondrial membrane potential, mitochondrial ion homeostasis and the generation and utilisation of the proton motive force. All modelled reactions and transport processes are depicted in our previous publication (Berndt et al.^13^). The metabolic model is coupled to a phenomenological model of hormonal signalling by glucagon and insulin affecting the short-term regulation of metabolic enzymes by reversible phosphorylation (see below). Uptake, metabolisation and generation of glucose, fructose, galactose, pyruvate, lactate, glycerol, ammonia, serine, alanine, glutamate, glutamine, fatty acids, ethanol, acetate, urea, acetoacetate, β-hydroxybutyrate, oxygen and VLDL particles are being described by the model.

### Short-term regulation of liver metabolism by hormones

The metabolism of the liver is strongly controlled by hormones, in particular insulin and glucagon.^15^ Glycolysis and gluconeogenesis as well as fatty acid synthesis and β-oxidation are inversely regulated by glucagon and insulin signalling via phosphorylation and de-phosphorylation of key regulatory enzymes. In the model, the plasma concentration of insulin and glucagon is directly translated into the phosphorylation state of interconvertible enzymes by a phenomenological sigmoid function (γ-function) also used in the work published by Bulik et al.^12^ Moreover, we used phenomenological transfer functions to compute the plasma concentrations of insulin and glucagon and of non-esterified fatty acids (NEFA) directly from the plasma level of glucose. This setting rests on the assumption that the release of insulin and glucagon from pancreatic islet cells is mainly controlled by the plasma glucose level and that high concentrations of glucagon and epinephrine stimulate the hormone-sensitive lipase (HSL) in adipose tissues, thus creating an inverse relationship between the plasma level of glucose and NEFA.

### LC-MS/MS proteome analysis

Murine liver samples were immediately frozen in liquid nitrogen and re-suspended in urea buffer (8 M urea, 100 mM Tris-HCl, pH 8.25) containing 100 µl of zirconium beads for protein extraction. Samples were homogenised on a Precellys 24 device (Bertin Technologies) for two cycles, 10 s at 6000 rpm. After centrifugation to remove beads and tissue debris, protein concentration was measured by Bradford colorimetric assay and 100 µg were taken for protein digestion. Leftover samples were frozen at −80 °C. The disulfide bridges of proteins were reduced in DTT 2 mM for 30 min at 25 °C and successively free cysteines alkylated in iodoacetamide 11 mM for 20 min at room temperature in the dark. LysC digestion was then performed by adding 5 µg of LysC (Wako Chemicals) to the samples and incubating it for 18 h under gentle shaking at 30 °C. After LysC digestion, the samples were diluted three times with 50 mM ammonium bicarbonate solution, 7 µl of immobilised trypsin (Applied Biosystems) was added and samples were incubated 4 h under rotation at 30 °C. Eighteen micrograms of the resulting peptide mixtures were desalted on STAGE Tips^16^ and the eluates dried and reconstituted to 20 µl of 0.5% acetic acid in water. Five microliters were injected in duplicate on a UPLC system (Eksigent Technologies) coupled to a LTQ Velos Orbitrap (Thermo Fisher Scientific), using a 240 min gradient ranging from 5 to 45% of solvent B (80% acetonitrile, 0.1% formic acid; solvent A = 5% acetonitrile, 0.1% formic acid). For the chromatographic separation 30 cm long capillary (75 µm inner diameter) was packed with 1.9 µm C18 beads (Reprosil-AQ, Dr. Maisch HPLC). On one end of the capillary nanospray tip was generated using a laser puller, allowing fretless packing. The nanospray source was operated with a spray voltage of 2.1 kV and an ion transfer tube temperature of 260 °C. Data were acquired in data dependent mode, with one survey MS scan in the Orbitrap mass analyzer (60,000 resolution at 400 *m/z*) followed by up to 20 MS/MS scans in the ion trap on the most intense ions. Once selected for fragmentation, ions were excluded from further selection for 30 s, in order to increase new sequencing events.

### Isolation and culture of primary murine hepatocytes and establishment of ASV-B cell lines

Primary hepatocytes from C57Bl/6 J were isolated as described earlier^17^ and maintained in Dulbecco’s modified Eagle’s medium (DMEM; high glucose; Thermo Fisher Scientific) containing 10% foetal bovine serum and 1% penicillin-streptomycin (Thermo Fisher Scientific) in collagen-coated flasks. All experiments were conducted within four days after primary cell isolation. HCC cells were isolated from 16-weeks-old ASV-B mice and cultivated in the medium described above. After an initial adaptation period of one to two months they started to proliferate and grow stably under cell culture conditions.

### Preparation and treatment of precision cut liver slices

Precision cut liver slices (200 µm thickness) were prepared from both normal and tumour-bearing murine livers based on a published protocol^18^ with slight modifications. Mouse livers were perfused with ice-cold University of Wisconsin organ preservation solution (UW), submerged in UW and kept on ice. Cylindrical cores with a diameter of 5 mm were prepared using a manual biopsy punch (Kai Medical Europe) and placed in a Krumdieck tissue slicer (model MD6000, Alabama Research & Development) containing ice-cold oxygenated Krebs-Henseleit buffer (KHB, Sigma–Aldrich). William’s E Medium (WME, Thermo Fisher Scientific) supplemented with 2.75 mg/ml D-glucose and 50 µg/ml gentamycin (Sigma–Aldrich) was used as the standard culture medium. To assess urea synthesis under stimulated conditions, DMEM (high glucose) with additional urea cycle substrates (2 µM ornithine and 10 µM NH_4_Cl (both Sigma–Aldrich)) was used. To determine the effect of complex I inhibition, PCLS were cultured in DMEM standard medium with or without 0.5 mM metformin (Cayman Chemical). Slices were incubated in a 6-well culture plate containing three slices per well and 3.5 ml medium. After 1 h of pre-incubation, slices were transferred to fresh medium and incubated for 24 h. Viability of the cultured tissue was confirmed by quantification of ATP with the Bioluminescence Assay Kit CLSII (Merck) and results were normalised to total protein content. Culture medium was collected and stored at −80 °C for later analysis of urea production. The concentration was determined by the University Hospital RWTH Aachen Central Laboratories applying standard diagnostic procedures.

### Proteomic data analysis

Proteomics raw data were analysed using the MaxQuant proteomics pipeline v1.4.1.2 and the built in Andromeda search engine^19,20^ with the mouse UniProt database (24,552 protein entries) and a common contaminants database (247 protein entries). Carbamidomethylation of cysteines was chosen as fixed modification, oxidation of methionine and acetylation of N-terminus were chosen as variable modifications. Two missed cleavage sites were allowed, and peptide tolerance was set to 7 ppm. The search engine peptide assignments were filtered at 1% FDR at both the peptide and protein level. The ‘match between runs’ feature was not enabled, ‘second peptide’ feature was enabled, while other parameters were left as default. For protein quantification LFQ intensities calculated by MaxQuant were used.^21^ The minimum LFQ ratio count was set to 2 and a MS/MS spectrum was always required for LFQ comparison of the precursor ion intensities; only unique and unmodified peptides were used for LFQ quantification, in order to keep the LFQ calculation isoform specific. Before comprehensive data analysis, data quality was evaluated using the in-house developed quality control software PTXQC.^22^

### Bioinformatic analyses

In total, 28 proteomic samples were analysed, coming for 14 mouse livers, with seven healthy and seven tumourous livers. Hence, the dataset comprised seven biological replicates for each condition, with two technical replicates per mouse liver. All bioinformatic analyses were performed in R. For each protein, technical replicates were averaged. Box and density plots were generated to assess the homogeneity of the sample distributions. Clustering was performed over complete cases of proteomics samples using the complete method and Euclidean distance (see pheatmap and hclust R packages) as well as principal component analysis. Differential analysis was performed using the LIMMA R package.^23^ This package assesses the significance of fold changes using parallel linear models sharing variance parameters. This method was originally developed for microarray data but turns out to be particularly suited for shotgun proteomics as it alleviates the scarcity of the measurement matrix by sharing the variance between proteins. Out of 2124 detected proteins, 1579 were used and tested for significance of their fold changes (detected in at least two samples in each condition). Nine hundred and thirty four proteins were found to have significant fold changes (FDR 5%). Metabolically relevant pathways for mouse were obtained from the GSKB R package,^24^ which propose a pre-processed metabolic pathway collection. The Piano R package was used to estimate the significance of the directional regulation of the pathways.^25^ The methods used to generate a consensual *p*-value in PIANO were: mean, median, sum, maxmean, stouffer, fisher, reporter, tailStrength, wilcoxon and PAGE. The FDR and *t*-values yielded by the LIMMA package were used as gene level statistics. The scripts and data can be accessed here: https://github.com/adugourd/Berndt_Egners_Mastrobuoni.

### Statistical analysis

All data are presented as mean ± standard error of the mean (SEM). Statistical analysis was performed by unpaired, two-tailed Students *t*-test using the GraphPad Prism 5.0 software (GraphPad Software) if not denoted otherwise. Differences were considered statistically significant at *p* < 0.05.

### Generation of tumour-specific instantiations of the kinetic model

For the generation of the tumour-specific kinetic model we used the quantitative proteomics data together with Hepatokin1, a fully kinetic model of the central carbon metabolism of the liver.^13^ We used the protein abundance data obtained from the murine samples to scale the activity (maximal velocity (Vmax) values) of the metabolic enzymes to generate tumour-specific kinetic models. These tumour-specific kinetic models were constructed by scaling the maximal activity of each enzyme and transporter in the network according to

1 $$v_{\max }^{{\mathrm{tumour}}} = v_{\max }^{{\mathrm{control}}}\frac{{E_{{\mathrm{tumour}}}}}{{E_{{\mathrm{control}}}}}$$

Here, E_tumour_ and E_control_ denote the LFQ intensity of the enzyme in the tumour and the control tissue. $v_{\max }^{{\mathrm{control}}}$denotes the maximal enzyme activity of the control (healthy liver) as defined in Hepatokin1.^13^ Equation1 is based on the fact that the LFQ intensity is proportional to the protein amount for each protein. While the proportionality constant depends on characteristic features of the peptides used as protein identifiers, the protein ratio is independent from this unknown constant as it cancels out in Eq.1. If the ratio of enzyme intensities in Eq. 1 could not be determined due to missing values of LFQ intensities, it was put to unity. This means that the corresponding enzyme is assumed to be unchanged compared to control conditions. The coverage was 67.5%.

## Results

### Proteome analysis of the central carbon metabolism in normal and malignant murine liver

**Fig. 1:**
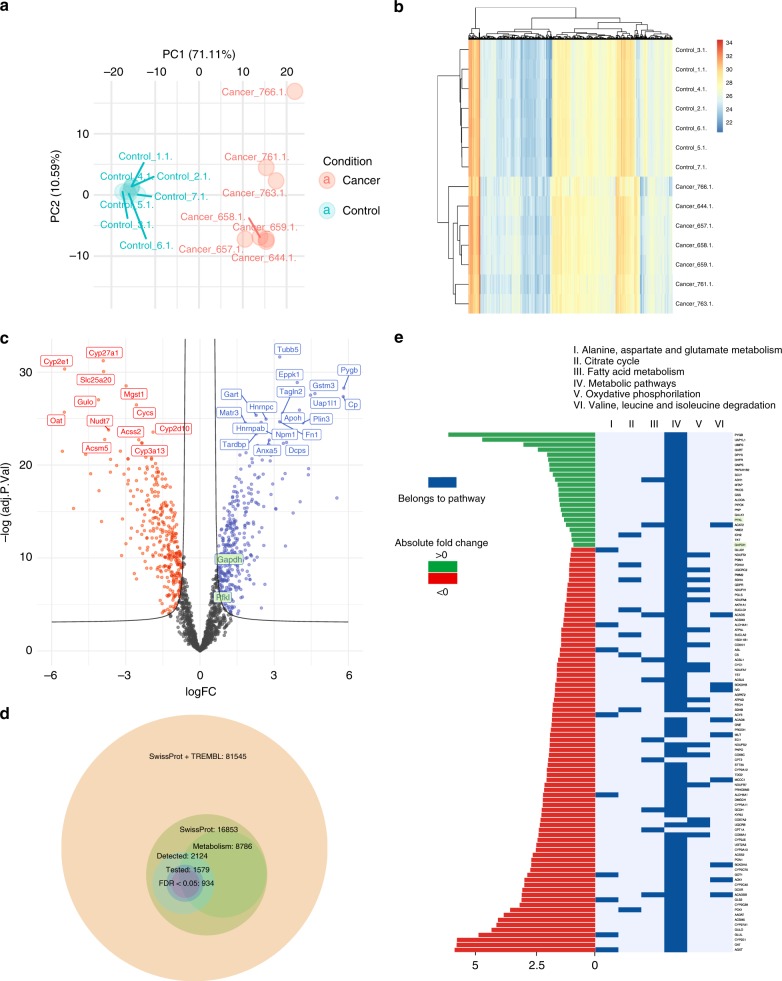
Detected metabolic enzymes in normal and HCC mouse liver and data quality control. **a** Principal Component Analysis (first two components, 71.11% + 10.59% of variance). Control (blue) and tumour (red) samples are well separated on the first component. **b** Clustering of the complete cases of proteomic samples. Control and tumour samples cluster together, respectively. **c** Volcano plot showing the log_2_ fold changes of proteins (HCC/control) with respect to the −log of FDR. The left side corresponds to proteins that are downregulated in tumour, while the right side corresponds to proteins that are upregulated in tumour tissue. Gapdh and Pfkl are highlighted in green. **d** Bubble plot showing the relations between the different protein sets considered in the study. Out of the 16,853 reviewed proteins present in the SwissProt database (of which 8786 are associated with metabolism), 2124 were identified by mass spectrometry. Significance of the fold changes between tumour and control could be estimated for 1579 proteins, of which 934 passed the threshold of 5% FDR. **e** Histomap showing the highly significant fold changes of 145 proteins (FDR ≤ 0.01%) associated with 6 significantly downregulated metabolic pathways (FDR ≤ 5%, protein sampling). Gapdh and Pfkl are highlighted in green.

In order to generate a detailed expression profile of enzymes of the central carbon metabolism, HCC samples from ASV-B mice as well as liver tissue from tumour-free control mice were analysed by a mass spectrometry-based shotgun proteomics approach.^20^ The proteomics experimental design consisted of 2 conditions (HCC and control (ctr)), seven biological replicates per condition and two technical replicates per biological replicate. In total, 2124 proteins were detected across the 28 samples. Each pair of technical replicates was averaged resulting in 14 biological samples (Fig. S1A, S1B). Principal Component Analysis showed that the variance of the mouse samples was well explained by their status (ctr or HCC). The control and tumour samples are clearly separated along the first principal component, which explains 71.11% of the variance of the dataset (Fig. 1a, S1C). Furthermore, the second component shows that the tumour samples display a greater inter-sample variability than the control samples, as expected given the aberrant regulation of tumours. A clustering of the proteomic profiles of the samples visually confirmed this, as the tumour profiles clearly display a greater heterogeneity than the controls (F test *p*-value: 3 × 10^-6^, Fig. 1b, S1D). The minimum correlation between pairs of biological replicates ranged from 0.65 to 0.95 (Fig. S1D). A differential expression analysis was performed between the control and tumour samples using linear models in order to estimate the significance of the changes in protein abundances (HCC/ctr, Fig. 1c and Methods). Out of 2124 proteins, 1579 were quantified in at least two samples in each condition (control and cancer) and the significance of their fold change was tested using the LIMMA R package. Out of 1579 tested proteins, 934 were associated with a false discovery rate (FDR) < 5% (Fig. 1d). The log_2_ fold change appears to be symmetrically distributed around zero. Gene set analysis was performed using the PIANO package^25^ in order to find consensual significant directional alteration of metabolism-related pathways, by incorporating fold-change directionality in the statistical enrichment analysis. The gene set collection originates from the GSKB R package, a mouse-tailored gene set resource similar to MSigDB. Many central metabolic pathways were found to be significantly downregulated (FDR ≤ 5%, Fig. S2), such as oxidative phosphorylation, citrate cycle and fatty acid metabolism (Fig. 1e). This observation suggests a robust reprogramming of cellular metabolism in the tumour samples towards downregulation of energy metabolism-related proteins.

In particular, we found that the majority of glycolytic enzymes such as Pfkl (the liver-specific 6-phosphofructokinase isoform) and Gapdh (glyceraldehyde-3-phosphate dehydrogenase) are significantly upregulated in HCC tissues (Fig. S3). Furthermore, the fructose-bisphosphate aldolase isoforms A and C show a more than two-fold higher expression, which, unlike isoform B, preferentially contribute to glycolytic rather than gluconeogenic metabolite turnover. In contrast, enzymes of other important metabolic pathways are downregulated such as pyruvate carboxylase, citrate synthase, succinate dehydrogenase, carnitine O-palmitoyltransferase 2, glutaminase (liver isoform), glutamine synthetase and ornithine carbamoyltransferase (Fig. S3).

In order to evaluate a potential contribution of infiltrating immune cells to the proteomics profiles we performed immunohistochemistry against CD45 and F4/80, established markers for leukocytes and macrophages, respectively. As can be seen in Fig. S4, abundance of both cell types is very low in murine HCC, suggesting a minor relevance of infiltrating immune cells for the obtained proteomics data.

### Prediction of tumour-specific metabolic capacity via mathematical modelling

**Fig. 2:**
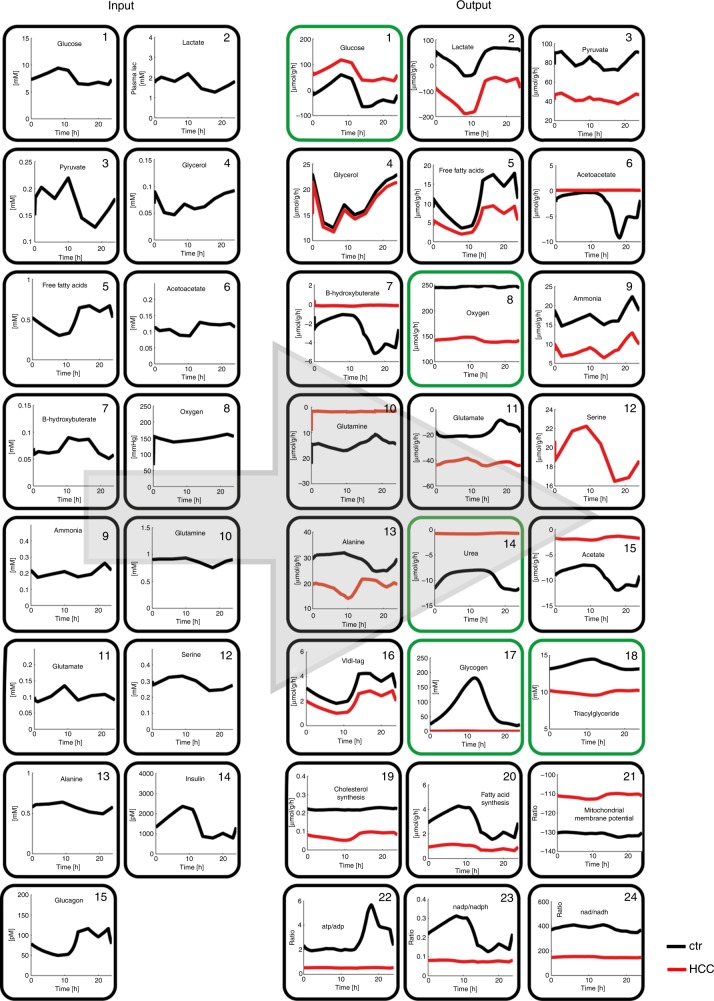
Simulated diurnal changes in the metabolic state of control and HCC liver. Input parameters (left panels) of the metabolic model are 24 h plasma profiles of metabolites and hormones. Model output (right panels) are the simulated diurnal profiles of 24 exchange fluxes and selected internal metabolites. Experimentally validated model predictions are highlighted in green.

Relative changes of protein abundances were mapped onto the maximal capacities (see methods) of the respective enzymes to generate a kinetic model of the central metabolism of murine HCC. To assess the functional consequences of alterations in metabolic enzyme expression, we applied the model to a typical 24 h physiological plasma concentration profile of exchangeable metabolites and the hormones insulin and glucagon. We used the plasma profile as model input and computed the diurnal variations in the concentrations of all model metabolites and fluxes for normal liver and murine HCC (Fig. 2). Compared with normal hepatocytes, the simulated metabolic response of murine HCC revealed a number of significant alterations (Fig. 2, right panels). The activity of glycolysis is strongly elevated while gluconeogenesis is almost completely suppressed. In line with these alterations, HCC is predicted to operate continuously as a strong lactate producer, while normal hepatocytes take up lactate. Fatty acid uptake, ß-oxidation of fatty acids, fatty acid and cholesterol synthesis are strongly diminished. Oxygen consumption is lower in HCC compared to normal liver. In addition, ammonia detoxification and urea synthesis in HCC are also clearly reduced (Fig. 2).

The model takes into account metabolic alterations that are caused by changes of enzyme abundance levels. In our computations we assumed that the regulation of enzyme activities in the tumour by hormone-dependent reversible phosphorylation is not altered compared to the control. As storage and degradation of glycogen is controlled by interconvertible enzymes, the discrepancies between predicted and measured glycogen content point to alterations in the signalling pathways of insulin and glucagon. A molecular resolved model of the underlying signalling pathways together with phospho-proteomics would be needed to improve the signalling part of the kinetic model. Another limitation of the current computational approach arises from the neglect of changes in the tissue architecture which may influence the exchange of metabolites between liver cells and sinusoid and thus may have a significant impact on metabolic functions.^26^

### Experimental validation of model predictions

**Fig. 3:**
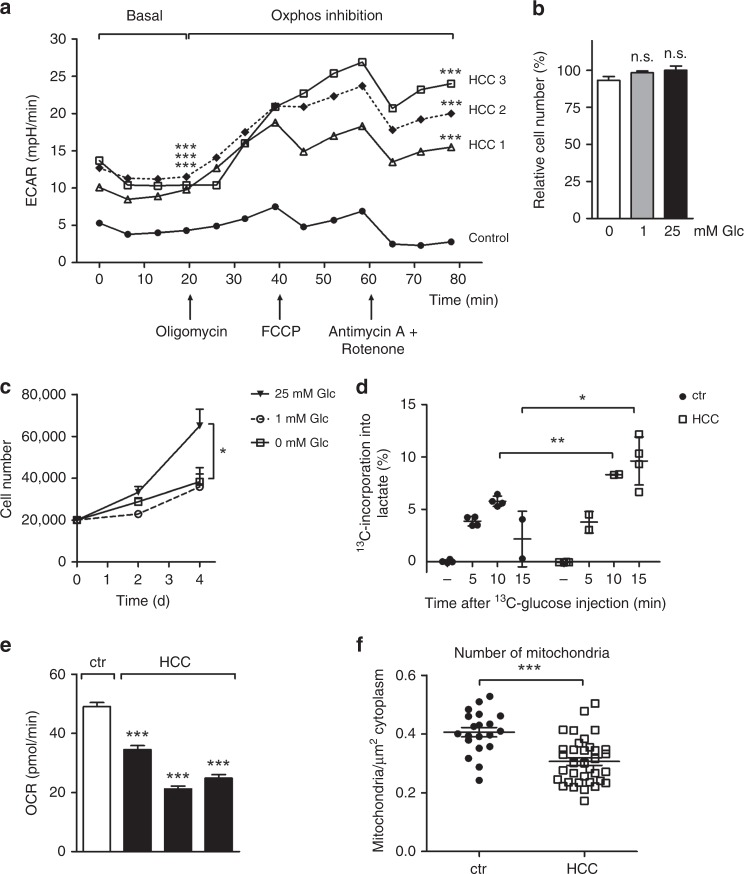
Experimental verification of predicted elevated glycolytic activity and reduced oxygen consumption in murine HCC. **a** Basal and post-respiratory chain complex inhibition extracellular acidification rates (ECAR) of isolated HCC cells and primary hepatocytes were measured. (*n* = 10). **b** Varying media glucose concentrations do not affect the survival of isolated primary hepatocytes (growing period of 72 h; *n* = 4). **c** Media glucose concentrations strongly affect the proliferation of isolated HCC cells. (*n* = 3). **d** In vivo pSIRM experiments reveal higher ^13^C-incorporation into lactate in tumours after i.p. injection of ^13^C-glucose. **e** Metabolic flux analyses on isolated cells show a lowered oxygen consumption rate of HCC cells (*n* = 10). **f** The number of mitochondria per µm² of cytoplasm was quantified by electron microscopy. *p *<* 0.05; **p < 0.005; ***p < 0.0001.

**Fig. 4:**
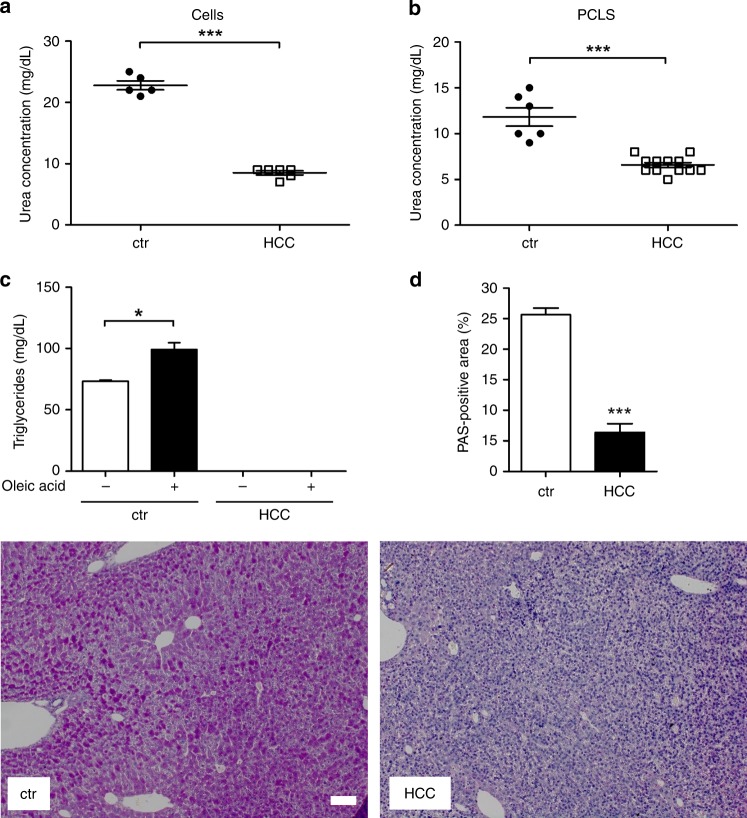
Experimental validation of urea production, intracellular triacylglyceride and glycogen storage. **a** Urea concentration in the supernatant of isolated hepatocytes and ASV-B cells and **b** PCLS. **c** Measurement of intracellular triacylglyceride in primary hepatocytes and isolated HCC cells without and after addition of oleic acid into the culture medium. (*n* = 3). **d** PAS staining for intracellular storage of glycogen on control and ASV-B liver sections as well as quantified PAS-positive area are depicted (ctr *n* = 4, HCC *n* = 5). Scale bar: 100 µm. *p < 0.05; ***p < 0.0001.

Next, we sought to perform a functional validation of selected model predictions. To validate the predicted changes of glycolysis (Fig. 2, output panel 1) and mitochondrial function (Fig. 2, output panel 8), extracellular flux analyses of normal primary hepatocytes (isolated from healthy C57Bl/6 J mice) and isolated HCC cells (from ASV-B mice) were performed. As can be seen in Fig. 3a, the extracellular acidification rate (ECAR) as a quantitative read-out of glycolytic activity is significantly elevated in HCC cells. Moreover, in contrast to hepatocytes, HCC cells are capable of further increasing the glycolytic rate after inhibition of mitochondrial respiratory chain complexes. In line with these results, we found that isolated primary hepatocytes are not affected by glucose restriction in the culture medium (Fig. 3b). In contrast, HCC cells isolated from ASV-B mice grew significantly slower in medium without glucose compared to standard medium (25 mM glucose; Fig. 3c). Pulsed stable isotope-resolved metabolomics (pSIRM) revealed higher label incorporation into lactate after intraperitoneal administration of ^13^C-glucose by ASV-B tumours compared to normal liver (Fig. 3d). This argues for elevated glycolytic activity of murine HCCs, well in line with the above outlined model prediction (Fig. 2, output panel 1). To test the mathematically predicted changes in oxygen uptake (Fig. 2, output panel 8), the oxygen consumption rate (OCR) was determined and found to be decreased in HCC cells (Fig. 3e). In addition, calculation of the OCR/ECAR-ratio showed that primary hepatocytes prefer oxidative phosphorylation over glycolytic energy production to a significantly greater extent than their HCC counterparts (Fig. S5A). To further analyse this, we quantified the cellular mitochondrial content with electron microscopy. As can be seen in Fig. 3f and S5B, these analyses indicated that the number of mitochondria is indeed significantly different between normal liver and murine HCC. Taken together, these functional assays display reduced mitochondrial activity in murine HCC and hence nicely confirm the model prediction shown in Fig. 2 (output panel 8). As outlined above, model calculations reveal a diminished capacity of HCC tumour tissue to synthesise urea in order to detoxify ammonia (Fig. 2, output panel 14). We validated this prediction by measuring the urea concentration in the cell culture supernatant and indeed found significantly less urea in the supernatant of ASV-B cells (Fig. 4a). Comparing the amount of urea produced by precision cut liver slices (PCLS) from normal and HCC liver supports this result (Fig. 4b). Additionally, we challenged the model prediction of impaired triacylglyceride production capacity in HCC cells (Fig. 2, output panel 18) by analysing the intracellular amount of triacylglycerides of cells without and after supplementation of the culture medium with oleic acid. Triacylglycerides were readily detectable in primary hepatocytes and their amount was increased by providing oleic acid. In contrast, no triacylglycerides were detectable in ASV-B cells regardless of the presence or absence of oleic acid (Fig. 4c). One key function of the liver is the intracellular storage of glycogen, which was predicted to be completely abolished in HCC tumours (Fig. 2, output panel 17). By performing Periodic acid–Schiff (PAS) staining for glycogen detection on sections from normal and ASV-B liver we in fact observed diminished staining in murine HCC tumours (Fig. 4d).

### Model-based predictions and functional validation of metformin treatment

**Fig. 5:**
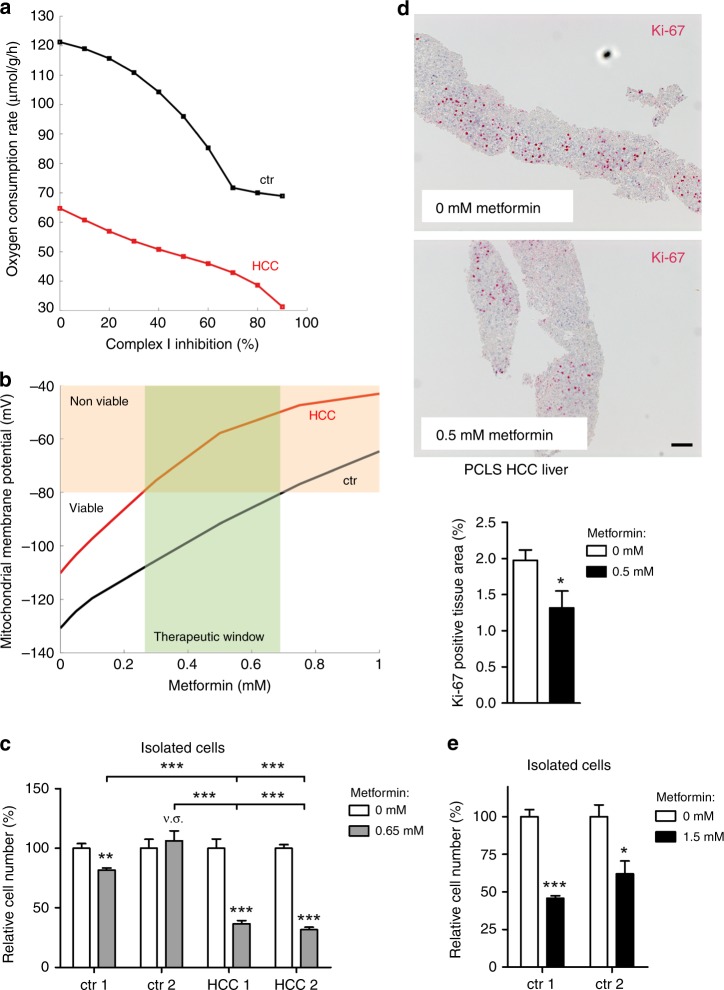
Mathematical sensitivity analysis identifies complex I inhibition as an effective anti-proliferative treatment for murine HCC. **a** Model predictions of oxygen consumption rate of control and HCC liver under the condition of complex I inhibition and **b** calculated mitochondrial membrane potential. **c** Relative cell numbers of primary hepatocytes and freshly isolated HCC cells was determined after treatment with metformin or control medium (significance was determined comparing results derived from treated versus non-treated cells, respectively, if not otherwise indicated; *n* = 4). **d** Ki-67 immunohistochemistry staining, and quantification demonstrates inhibition of proliferation in HCC tissue caused by metformin treatment (0 nM *n* = 6, 0.5 mM *n* = 8). Scale bar: 100 µm. **e** Isolated hepatocytes were treated with 1.5 mM metformin or control medium. The relative cell number was measured (*n* = 4). *p < 0.05; **p ≤ 0.005; ***p < 0.0001.

We hypothesised that the reduced capacity of oxidative phosphorylation in HCC can be exploited for cancer therapy by serving as a metabolic target to selectively impair HCC metabolism while leaving healthy liver intact. This was further strengthened by modelling the oxygen consumption as a function of mitochondrial complex I activity, demonstrating consistently lower values of tumour compared to control liver (Fig. 5a). The antidiabetic drug metformin has been shown to inhibit neoplastic growth by multiple mechanisms,^27^ one of them being complex I inhibition.^28^ In addition, it was shown that metformin acts as non-competitive inhibitor of mitochondrial glycerol-3-phosphate dehydrogenase (G3pdh), explaining its antidiabetic properties.^29^ Using the reported inhibition concentrations of metformin of 0.5 mM for complex I^28^ and 0.055 mM for G3pdh,^29^ we simulated the effect of metformin on HCC and healthy liver. We put the external conditions to their mean value over one day and varied the metformin concentration from 0 to 1 mM. Figure 5b depicts the mitochondrial membrane potential of healthy liver and HCC as a function of the metformin concentration. As mitochondria induce apoptosis in response to energy depletion (once the mitochondrial membrane potential [MMP] rises above ~−80 mV), the simulations predict damage to the liver tumours already at 0.27 mM metformin, while healthy hepatocytes remain viable up to metformin concentrations of 0.7 mM. We functionally validated these results by treating freshly isolated HCC cells and primary hepatocytes with metformin. In line with model predictions, exposure to 0.65 mM metformin only mildly affected healthy liver cells derived from control mouse 1, whereas ctr2 cells were completely unaffected (Fig. 5c). In contrast, treatment with 0.65 mM metformin resulted in robust growth inhibition of HCC cells. A significant blockade of proliferation was also observed after treatment of HCC precision cut liver slices (PCLS) with metformin (Fig. 5d). In compliance with model predictions, exposure to higher metformin concentrations (1.5 mM) strongly decreased the viability of primary hepatocytes (Fig. 5e).

## Discussion

The fundamental metabolic reprogramming processes that tumour cells undergo to support growth and survival have received widespread attention in recent years and are now considered as an emerging hallmark of cancer.^8,30^ However, exploitation of metabolic vulnerabilities to identify effective and specific anti-cancer agents remains challenging. The advancement of analytical technologies like shotgun proteomics opened the way for global snapshots of the molecular makeup of healthy tissues and tumours.^31,32^ The increased sensitivity of these technologies together with improved data reproducibility enable for the first time to map the biochemical network in its totality.^9^ However, due to (i) enormous plasticity and dynamics, (ii) multi-level regulatory mechanisms and (iii) a highly complex network of reactions, cellular metabolic processes are difficult to study. Mathematical models are useful tools to unravel this complexity, and various tools have been established to develop and analyse genome-scale metabolic models cells.^33^ Using omics data to scale cell-wide network models of metabolism has led to the identification of novel drug targets and biomarkers.^34^ Naturally, mathematical models are always simplifications of multi-level biological phenomena; however, hitherto published metabolic models of liver metabolism specifically lack important regulatory aspects, e.g. hormonal influences and allosteric parameters. In addition, they are very often not based on data that have been established experimentally but on information solely extracted from published literature.

Here, we used a comprehensive kinetic model of the central carbon and lipid metabolism of hepatocytes that incorporates not only the metabolic reaction network but also enzyme regulation by allosteric effectors and by reversible phosphorylation due to changing insulin and glucagon signalling as in Berndt et al.^13^ The influence of fluctuating nutrient (like glucose and glutamine) and oxygen concentrations within a physiological range are also taken into account. As demonstrated by us earlier, these parameters are at least equally important for modelling the metabolic performance as the changes in enzyme abundance (see Bulik et al.^12^). Applying the model to the central metabolism of HCC, we took advantage of the fact that HCCs originate from hepatocytes,^35^ i.e. metabolic enzymes in normal and malignant cells only differ in their expression level. This enabled us to re-parameterise the hepatocyte model by scaling the enzyme activities between HCC and normal hepatocytes according to the observed changes of protein abundances that we assessed by quantitative mass spectrometry. It has to be taken into account that relative abundances of cell types other than HCC cells also contribute to the determined protein expression profiles to a certain extent. Immunohistochemistry staining, however, demonstrated negligible infiltration of immune cells in HCC tumour nodules. We therefore assume that the contribution of other cell types to the final proteomics datasets is rather small. This assumption is substantiated by the fact that experimental results obtained from isolated HCC cells were well in line with calculated model predictions. The present study proves that our approach is able to transform mass spectrometry protein data into biologically and clinically meaningful metabolic predictions about the HCC tissue turnover activity of various metabolites. Comparison of simulated normal and HCC liver metabolic performance reveals fundamental differences. We validated several of the calculated model output parameters successfully using different experimental approaches. Glycolytic and respiratory activity were determined by measuring the extracellular acidification and oxygen consumption rates, confirming that HCC cells show increased glycolytic and reduced mitochondrial activity. These findings are well in line with earlier reports using transcriptomics, metabolomics or enzyme activity measurements on different murine HCC models.^36–38^ Increased glycolytic activity is consistently found in independent analyses of human HCC tissue with different omics approaches and non-invasive imaging (NMR spectroscopy), pointing towards the Warburg effect as a metabolic hallmark of human liver cancer.^39–41^

The kinetic model predicted HCC-specific alterations of urea and triacylglyceride synthesis as well as glycogen storage, all of which represent key functions of normal liver. The functionality of the urea cycle in HCC tissue has been under debate for quite some time. It had been established rather early on that HepG2 cells, one of the most widely used human HCC cell lines, harbour a defective urea cycle and it was later shown that this is due to ornithine transcarbamylase and arginase I deficiency.^42^ Butler et al. showed that the human hepatocellular carcinoma cell lines Huh-7, HepG2 and LH86 do not express the urea cycle enzyme carbamoyl phosphate synthetase 1.^43^ A comprehensive microarray analysis of 521 patient-derived HCC samples revealed suppression of urea cycle-associated enzymes.^44^ On the other hand, arginase I and carbamoyl phosphate synthetase (Cps) were found overexpressed in human HCC and their detection via immunohistochemistry was suggested to improve the histopathological diagnosis of HCC.^43,45^ Our approach now reveals—for the first time—reduced urea cycle activity in a murine HCC model, nicely confirming that systematic integration of protein expression data is a prerequisite to comprehend metabolic pathway activity.^46^ The analysis of HCC-specific changes of lipid metabolism has received a lot of attention recently (over 20 studies using human samples and different omics approaches were published in the last 10 years^47^). The reported results are very heterogeneous, precluding the identification of an HCC-specific lipid metabolism pattern. If anything, activation of fatty acid catabolism (most importantly β-oxidation) could be considered a hallmark of HCC-specific lipid metabolism as it was reported by the majority of publications.^47^ Of note, the activity of anabolic lipid metabolism pathways in HCC has received significantly less attention. Our approach of combining quantitative proteomics with mathematical modelling predicted reduced activity of several anabolic lipid pathways in HCC, e.g. synthesis of triacylglycerides, cholesterol and fatty acids as well as VLDL secretion. We were able to functionally validate the calculated reduction of triacylglyceride synthesis, underscoring the eligibility of the comprehensive kinetic model to forecast alterations of lipid metabolism in HCC. In summary, the results of all validation experiments show striking consistency with the calculated model simulations indicating that our kinetic model is indeed a powerful tool to reproduce HCC metabolism in a reliable manner.

The feasibility of the translational application of our kinetic model to estimate and evaluate the performance of therapeutic agents with prediction of possible adverse effects is demonstrated by calculating the outcome of metformin treatment on HCC viability. The antidiabetic drug metformin received a lot of attention in recent years after it was reported to reduce cancer risk and mortality in diabetic patients.^48^ Metformin was subsequently shown to exert anti-tumour effects against established human HCC cell lines and in HCC xenografts in nude mice.^49^ Our results confirm the cell line data reported by Miyoshi et al. and furthermore show that primary hepatocytes are not affected by metformin at the doses found to inhibit HCC cells. These results demonstrate the versatility of our kinetic model as, in addition to depicting metabolic activities, it is able to predict metabolic vulnerabilities. We propose that the herewith outlined experimental approach will also be able to identify innovative targets for the therapy of human HCC. One could even envision an application in a clinical setting where mathematical modelling of biopsy-derived quantitative mass spectrometry-based proteomics data could assist the design of a personalised therapy tactic. Future work with patient samples will need to thoroughly test this hypothesis before a potential exploitation for HCC therapy.

## Supplementary information

supplemental material, figures and figure legends in one file

executable SBML file of Hepatokin1

## Data Availability

The mass spectrometry results (project accession: PXD011162) have been deposited in the EMBL-EBI PRIDE archive (https://www.ebi.ac.uk/pride/archive/login).
