# Supplementary material for: Kinetic modelling of quantitative proteome data predicts metabolic reprogramming of liver cancer
Source: Br J Cancer. 2019 Dec 10;122(2):233–44. doi: 10.1038/s41416-019-0659-3 (PMC7052204; doi:10.1038/s41416-019-0659-3)
Supplement: Supplementary file 1 — supplemental material, figures and figure legends in one file [file 41416_2019_659_MOESM1_ESM.docx]

**Supplementary Data**

**Supplementary Methods**

***In vivo* pulsed stable isotope resolved metabolomics**

Frozen liver tissue was grounded using mortar and pestle and the powdered tissue was directly resolved in pre-cooled extraction buffer (-20°C, chloroform/methanol/water (2:5:1 vol/vol/vol). 50 mg tissue were resolved in 1 ml extraction buffer including the internal standard cinnamic acid (Sigma-Aldrich) 2 µg/ml. Samples were shaken for 1 hour at 4°C, subsequently 500 µl water were added, samples shaken for 30 minutes at 4°C, centrifuged for 10 minutes at 10,000 g and polar and lipid phase collected and dried overnight in a speed vac.

**Metabolomics measurements and data analysis**

Dried polar extracts were resolved in 20 µl pyridine including 40 mg/ml Methoxamine hydrochloride (Sigma-Aldrich) and shaken for 1 hour at 30°C. Subsequently, 80 µl Methyl-N-(trimethylsilyl) trifluoroacetamide (MSTFA, Fluka) was added and samples were shaken for 90 min at 37°C. Samples were centrifuged (10 min at 10,000 g) and transferred into glass vials (Chromacol). 1 µl of the samples was injected into an Agilent gas chromatograph 6890 equipped with a temperature controlled injection system (ALEX, Gerstel). Data were acquired using a GC-ToF-MS (Pegasus III, Leco), processed using the vendor software Chromatof (Leco) and analyzed using the MetMax software [^1^](#_ENREF_1). Identification of metabolites was performed using pure chemicals (Sigma-Aldrich), quantification of metabolites and calculation of isotope incorporation were performed as described [^2^](#_ENREF_2).

**Cellular metabolic rate measurements**

Primary hepatocytes and ASV-B cells were seeded 24 hours ahead of analysis (7,000 cells per well in collagen-coated XF96 cell culture plates, Seahorse Bioscience) in standard medium. One hour before experimental start the cells were supplied with XF Base medium (Seahorse Bioscience) supplemented with 2 mM glutamine and 10 mM glucose. Oxygen consumption rate (OCR) and extracellular acidification rate (ECAR) were measured using the XF^e^96 Extracellular Flux Analyzer (Seahorse Bioscience) according to the manufacturer’s instructions.

**Urea quantification**

For the analysis of urea synthesis, 500,000 ASV-B cells or primary hepatocytes were seeded in 6-well plates with 2 ml of growth medium (DMEM high glucose, 10% FCS and 1% penicillin/streptomycin). The medium was replaced 18 hours later and the cells were incubated for further 24 hours. 1.5 ml supernatant was collected and centrifuged at 1,000 g for 5 minutes to separate cells and debris. The remaining supernatant (1.2 ml) was transferred to a new tube and the samples were stored at -80°C until further use. Urea measurements were conducted by the University Hospital RWTH Aachen Central Laboratories applying standard diagnostic procedures.

**Intracellular triglyceride measurement**

Intracellular triglycerides in ASV-B cells and primary hepatocytes were determined using the Triglycerides liquicolor^mono^ assay (HUMAN Diagnostics). Briefly, 400,000 cells per well were seeded in 1 ml standard medium with 10% FCS and 1% penicillin/streptomycin in 6 well plates and left for 4 hours to attach. Subsequently, the medium was replaced by DMEM (high glucose) supplemented with 0.5% FBS for 16 hours to starve the cells. Then the cells were either left untreated or stimulated with 300 µM oleic acid-albumin (Sigma-Aldrich) for 24 hours. The next day, the cells were washed with PBS once and harvested in 100 µl homogenization buffer (10 mM Tris, 2 mM EDTA, 250 mM sucrose at pH 7.5). Homogenization was conducted by sonication (6 impulses with 10% intensity by sonication, Sonoplus Type UW3100, Bandelin). Cell debris was separated by centrifugation. The assay was conducted according to the manufacturer’s instructions.

**Histochemistry**

PAS, Ki-67 and staining against the immune cell markers CD45 (BD Biosciences) and F4/80 (Thermo Fisher Scientific) as well as counterstaining with hematoxylin were performed according to routine histochemistry protocols. The ImageJ software (NIH) was used in order to calculate PAS- or Ki-67-positive areas (data normalization was conducted using the total analyzed area).

**Transmission electron microscopy (TEM)**

TEM was performed by the Core Facility for Electron Microscopy of the Charité Berlin as outlined in detail before [^3^](#_ENREF_3).

**Supplementary Figures and Figure Legends**

**
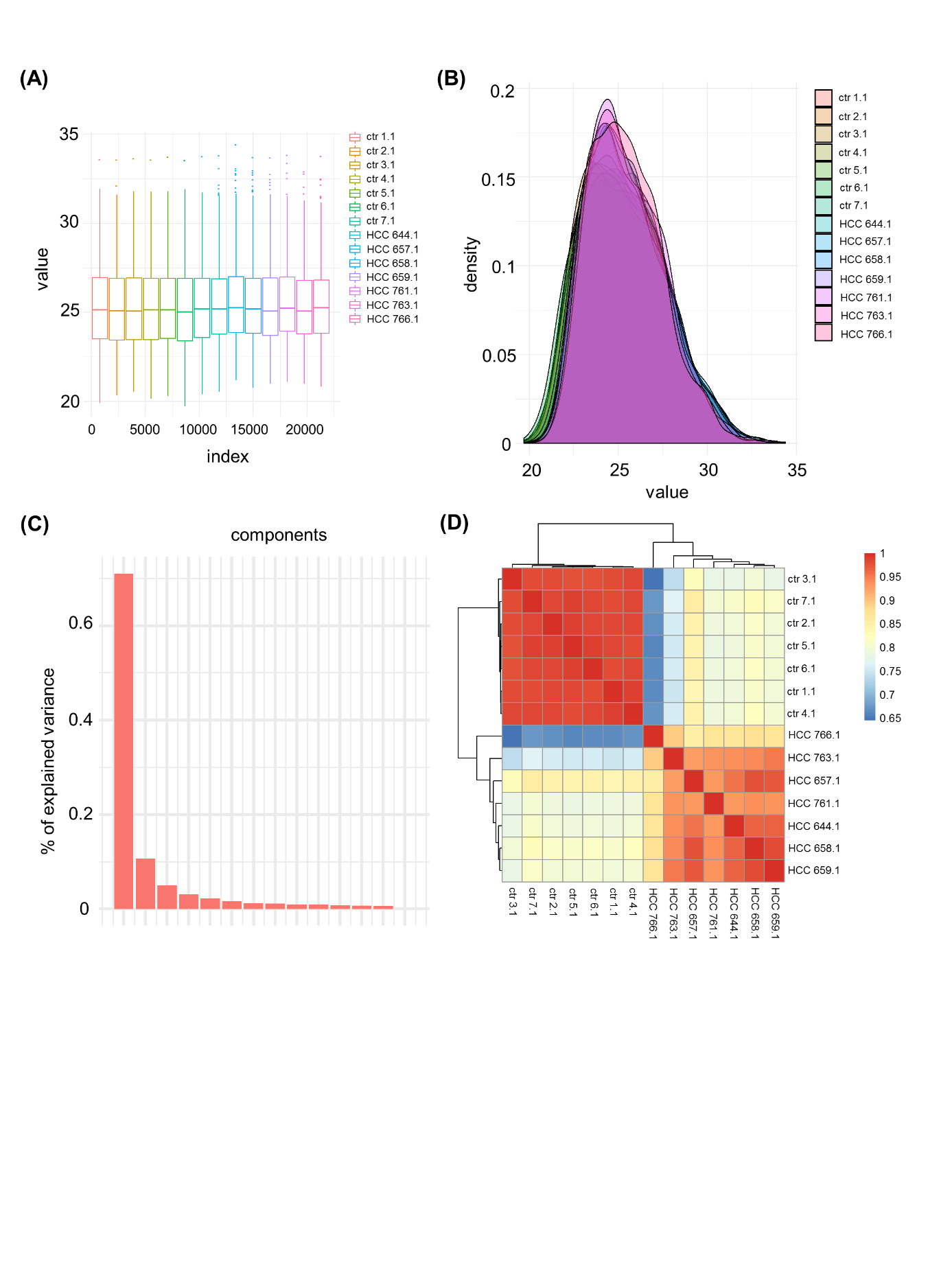
**

**Figure S1. Detailed quality control of the mass spectrometry-based proteomics analysis.** (A) Boxplot of the 14 biological replicate samples. Y axis represents the log_2_ transformed LFQ.intensity of the proteins. (B) Density plot of the log_2_-transformed LFQ intensities of proteins from 14 biological replicate. The box plot and density plot together show that the log_2_-transformed LFQ intensities follow homogeneous normal-like distributions. (C) Boulder plot depicting the proportion of the explained variance of the Eigen values of the Principal Component Analysis. (D) Correlation heatmap of the 14 biological replicates. Correlation coefficients are calculated using the Pearson method, and the clustering was performed with the complete method and Euclidean distances.

**
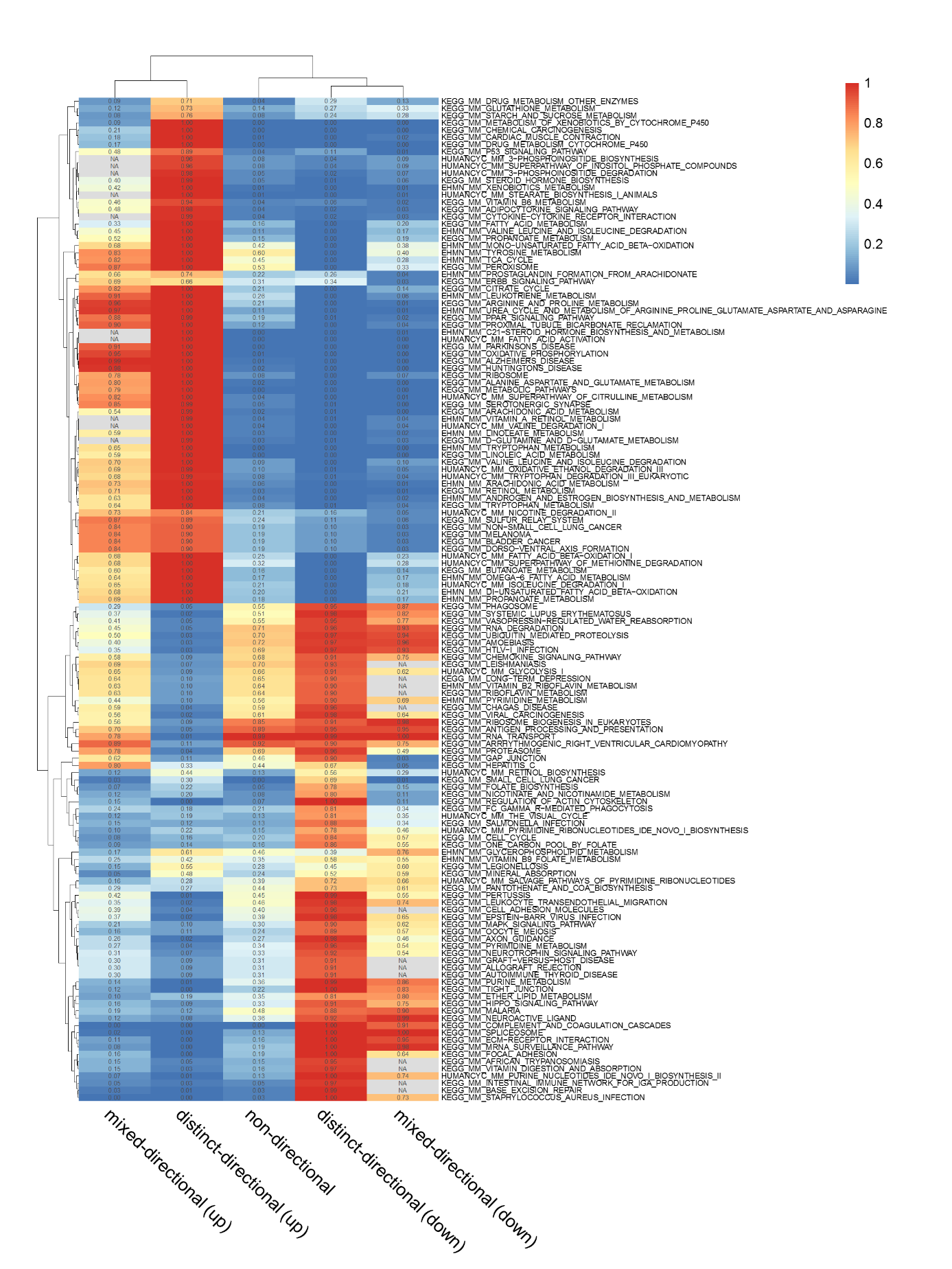
**

**Figure S2. Heatmap of proteomics data.** Five regulation classes of 139 significantly regulated metabolically relevant mouse pathways (PIANO consensus score > 50) are presented. Significance of the pathways are shown as FDR.

**
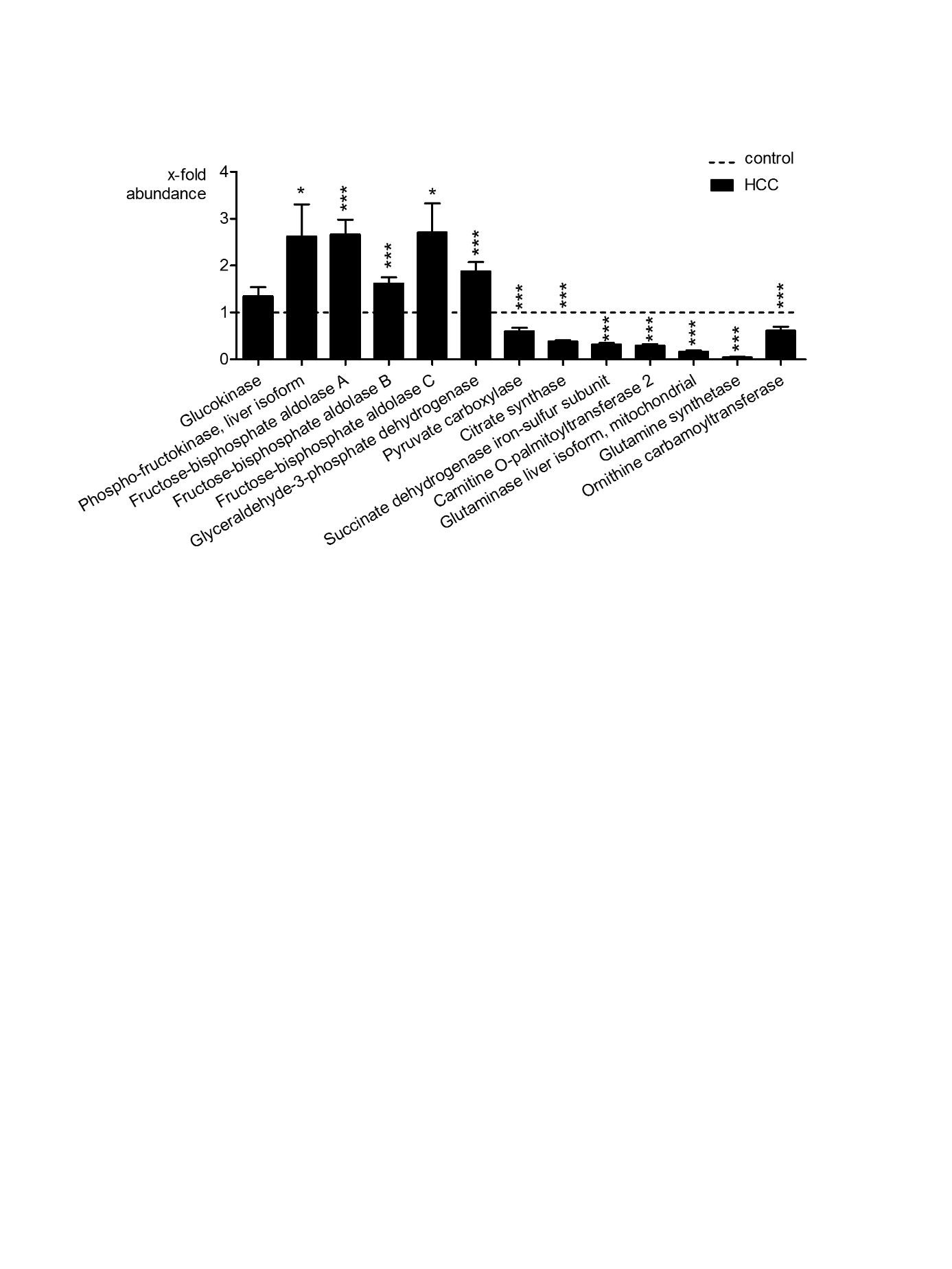
**

**Figure S3.** **Quantification of protein expression of selected metabolic enzymes.** Expression based on mass-spectrometry proteomics measurements is shown (n = 5 to 7; significance was determined comparing protein abundance in HCC and control tissue). *, *p* < 0.05, ***, *p* < 0.0001.

**
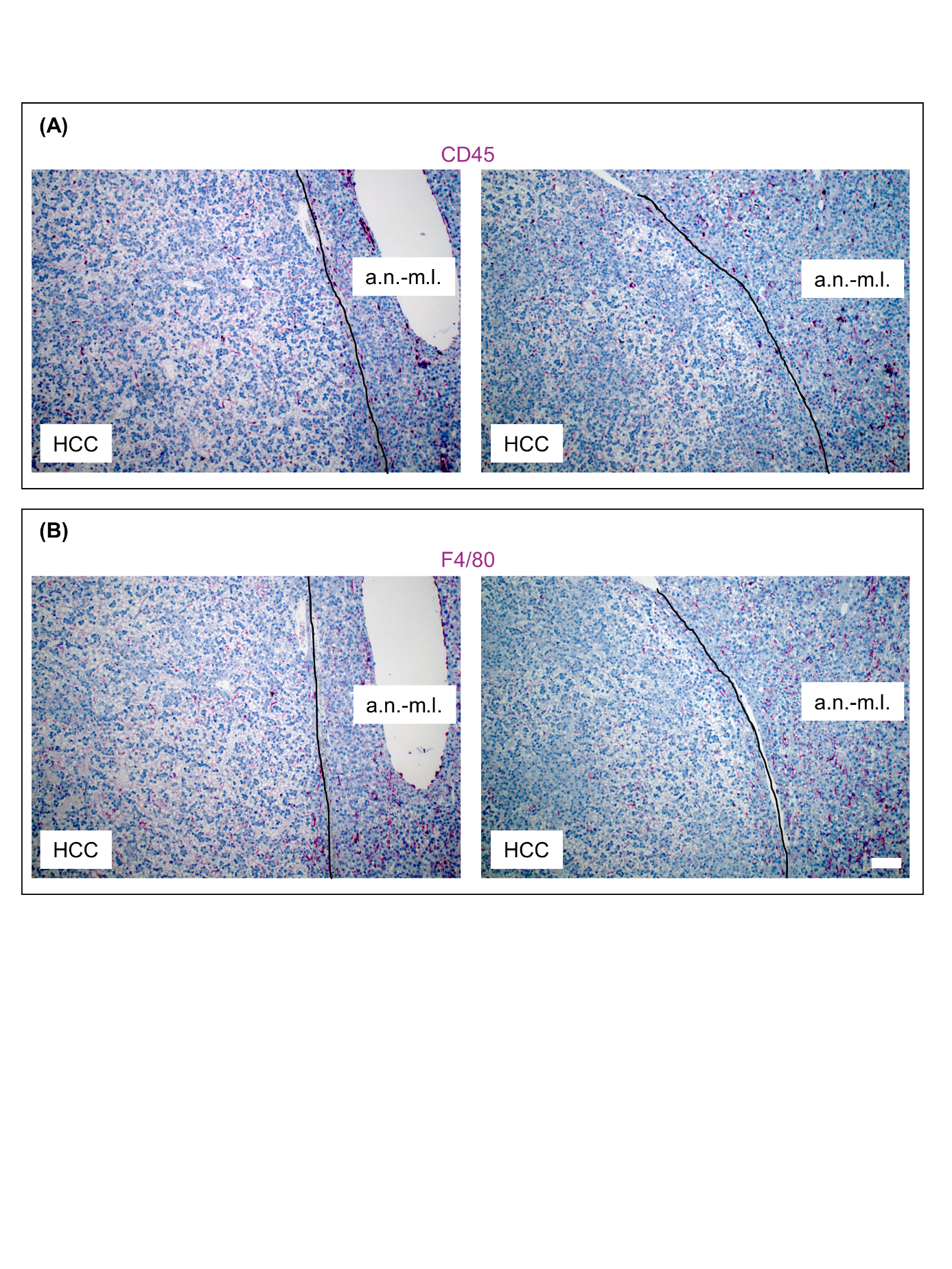
**

**Figure S4. Analysis of tumor-associated inflammation.** Immunohistochemistry staining for (A) the leukocyte marker CD45 and (B) the macrophages marker F4/80 on murine HCC tissue sections including adjacent non-malignant liver (a.n.-m.l.). Scale bar: 100 µm.

**
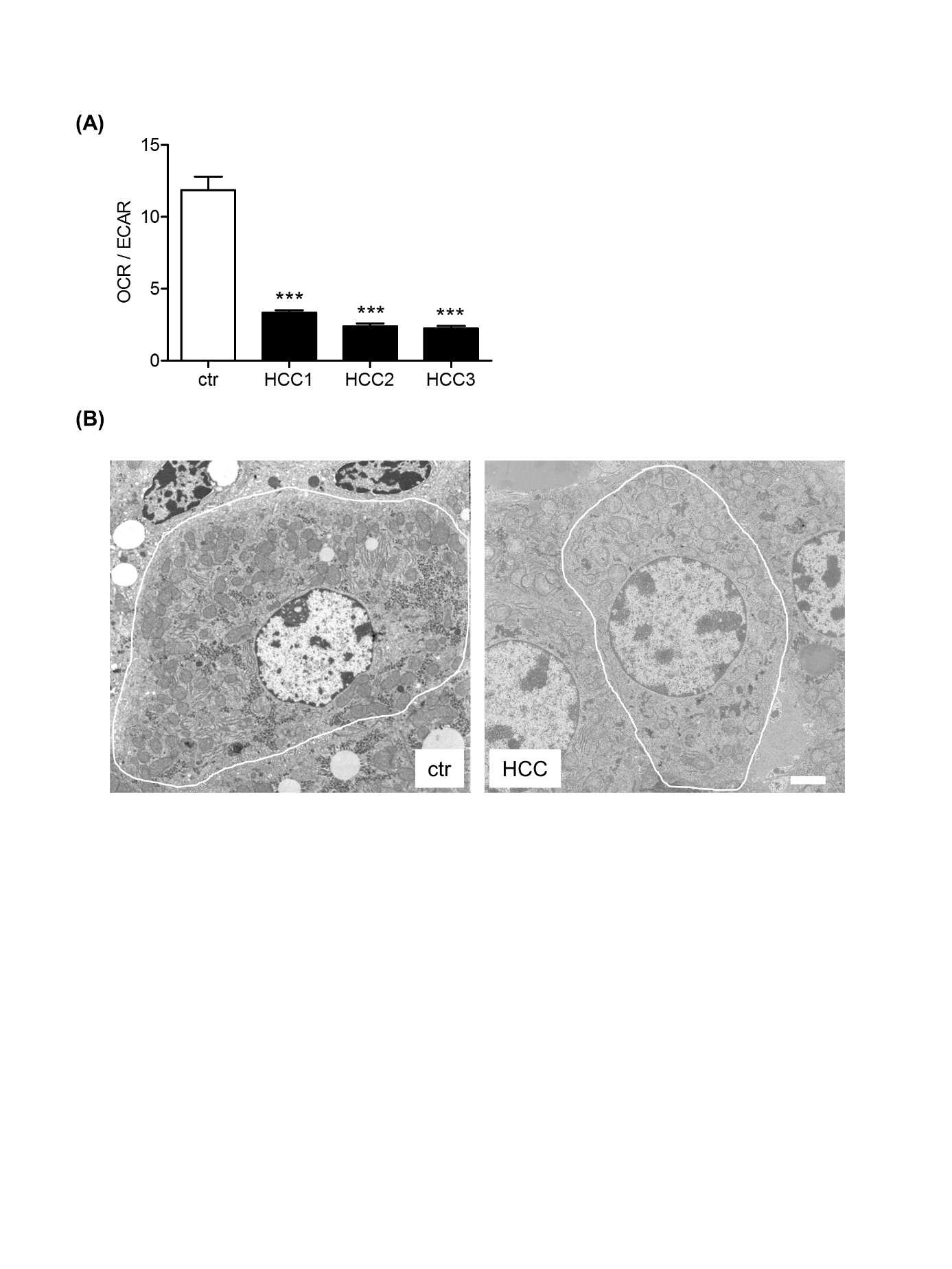
**

**Figure S5. Comparison of mitochondrial function and number.** (A) OCR/ECAR-ratio in control and HCC cells (n = 10). ***, *p* < 0.0001. (B) Representative electron microscopy images of normal (ctr) and ASV-B liver (HCC) sections. Scale bar: 2 µm.

**Supplementary References**

1. Kempa S, Hummel J, Schwemmer T, Pietzke M, Strehmel N, Wienkoop S *et al.* An automated GCxGC-TOF-MS protocol for batch-wise extraction and alignment of mass isotopomer matrixes from differential 13C-labelling experiments: a case study for photoautotrophic-mixotrophic grown Chlamydomonas reinhardtii cells. *Journal of basic microbiology* 2009; **49**(1): 82-91; e-pub ahead of print 2009/02/12; doi 10.1002/jobm.200800337.

2. Pietzke M, Zasada C, Mudrich S, Kempa S. Decoding the dynamics of cellular metabolism and the action of 3-bromopyruvate and 2-deoxyglucose using pulsed stable isotope-resolved metabolomics. *Cancer & metabolism* 2014; **2**: 9; e-pub ahead of print 2014/07/19; doi 10.1186/2049-3002-2-9.

3. Theilig F, Bostanjoglo M, Pavenstadt H, Grupp C, Holland G, Slosarek I *et al.* Cellular distribution and function of soluble guanylyl cyclase in rat kidney and liver. *Journal of the American Society of Nephrology : JASN* 2001; **12**(11): 2209-2220; e-pub ahead of print 2001/10/25.
